# Effects of Permissive Hypercapnia on Laparoscopic Surgery for Rectal Carcinoma

**DOI:** 10.1155/2019/3903451

**Published:** 2019-10-07

**Authors:** Lei Wang, Lina Yang, Jing Yang, Shiqiang Shan

**Affiliations:** Department of Anesthesiology, Cangzhou Central Hospital, Cangzhou 061000, China

## Abstract

**Background:**

Permissive hypercapnia has been recommended during the treatment of chronic diseases; however, there are insufficient clinical data to investigate the feasibility of permissive hypercapnia in relatively long-term surgeries such as laparoscopic surgery for rectal carcinoma. This prospective study is aimed at investigating the efficacy and safety of permissive hypercapnia under different CO_2_ pneumoperitoneum pressures during the laparoscopic surgery for rectal carcinoma.

**Methods:**

A total of 90 patients undergoing laparoscopic surgery for rectal carcinoma were recruited from July 2016 to March 2017. They were randomly assigned to high hypercapnia group (*n* = 30), low hypercapnia group (*n* = 30), or control group (*n* = 30), whose PaCO_2_ levels were maintained at 56-65 mmHg, 46-55 mmHg, or 35-45 mmHg, respectively. The primary endpoint was peak pressure. Plateau pressure, dynamic compliance, arterial blood analysis, and hemodynamic measures were collected as secondary outcomes. Adverse events were monitored.

**Results:**

High hypercapnia group were reported to be associated with significantly lower peak pressure and plateau pressure, but higher dynamic compliance compared to low hypercapnia and control group (all *P* < 0.01). Moreover, patients in the high hypercapnia group had higher postoperation oxygenation index values compared to those in the low hypercapnia and control group (all *P* < 0.01). There is no significant difference in the pH, Spo_2_, MAP, heart rate, and adverse events among the three groups.

**Conclusion:**

Permissive hypercapnia with a PaCO_2_ level of 56-65 mmHg was able to improve respiratory function after laparoscopic surgery in rectal cancer patients.

## 1. Introduction

Colorectal cancer is the third most common cancer, accounting for almost 1.4 million new cases and 694,000 deaths in 2012 [1]. In particular, retrospective data from China showed that the proportion of rectal cancer was 59.4%-71.0% of the total number of colorectal cancers [2].

The mainstay treatment for rectal cancer remains surgical resection. Recently, laparoscopic surgeries have increased manifold for rectal cancer owing to favorable short-term outcomes, such as less pain, reduced blood loss, and improved recovery time as compared to surgeries performed by traditional techniques [3, 4]. During laparoscopic procedures, carbon dioxide (CO_2_) is commonly used to insufflate the abdomen so as to facilitate the surgical view. Meanwhile, the accumulation of CO_2_ may lead to elevated arterial carbon dioxide partial pressure (PaCO_2_) and hypercapnia. Permissive hypercapnia is a ventilation strategy to allow for an unphysiological PaCO_2_ to permit lung protective ventilation with lower tidal volumes. Current guidelines recommend the concept of lower tidal volume ventilation and permissive hypercapnia for patients with sepsis, acute respiratory distress syndrome (ARDS), or acute or chronic respiratory failure [5, 6]. However, there are insufficient clinical data to investigate the feasibility of permissive hypercapnia in relatively long-term surgeries such as laparoscopic surgery for rectal carcinoma.

The present study is aimed at investigating the efficacy and safety of permissive hypercapnia under different CO_2_ pneumoperitoneum pressures during laparoscopic surgery for rectal carcinoma.

## 2. Materials and Methods

### 2.1. Patients

This prospective study was conducted at the Cangzhou Central Hospital, China, from July 2016 to March 2017. A total of 90 patients aged ≥ 18 years with American Society of Anesthesiologists (ASA) physical status II-III classification were enrolled in the current study. All patients had a pathological or cytological diagnosis of adenocarcinoma of the rectum. Patients with a second primary malignancy, severe cardiac dysfunction, intracranial disease, mental disorder, visual or auditory dysfunction, prior therapies for rectal cancer, or difficulties in laparoscopic surgery (e.g., severe chronic obstructive pulmonary disease, acute inflammatory bowel disease, obesity, and pregnancy) were excluded from the study. In addition, patients with a surgery more than 5 hours or who were converted to laparotomy during laparoscopic surgery were excluded.

This study was approved by the institutional review board of Cangzhou Central Hospital, China. All procedures followed were in accordance with the Helsinki Declaration of 1964, as revised in 2013. All patients provided written informed consent.

### 2.2. Randomization

All patients were randomly assigned into a high hypercapnia group (*n* = 30), low hypercapnia group (*n* = 30), or control group (*n* = 30) using the sealed envelope system, whose PaCO_2_ levels were maintained at 56-65 mmHg, 46-55 mmHg, or 35-45 mmHg, respectively. Randomization was stratified according to gender, ASA physical status, and clinical stage. Patients and individuals assessing outcomes were not masked to treatment assignment.

### 2.3. Procedures

General physical examination and assessment for the airway were performed before the operation. Preoperative fasting of a minimum of 8 hours was ensured before the operation in all cases. All patients were transferred to the operating room and subjected to percutaneous radial artery cannulation to monitor mean arterial pressure (MAP) and to analyze blood gas levels. These patients were given injections of lidocaine (1.0-1.5 mg/kg), fentanyl (2.0-4.0 *μ*g/kg), rocuronium (0.6 mg/kg), and propofol (1.0-2.0 mg/kg). After induction, a left or right double-lumen endobronchial tube was inserted. The patients were treated with propofol (2.0-4.0 mg·kg^−1^·h^−1^) and remifentanil (0.1-0.2 *μ*g·kg^−1^·min^−1^) for the maintenance of anesthesia.

Pneumoperitoneum was created by skilled surgeons with CO_2_ gas using a Veress needle introduced through a periumbilical puncture. It was executed initially at a slow flow (1 l/min) and then faster flow (3-5 l/min) to avoid a vasovagal reaction. The PaCO_2_ was maintained at 56-65 mmHg for the high hypercapnia group, 46-55 mmHg for the low hypercapnia group, and 35-45 mmHg for the control group throughout the procedure.

The surgical operating principles were the same for the three groups, following strict rules for achieving a tumor-free status. All procedures were required to comply with the principles of total mesorectal excision (TME) or partial mesorectal excision (PME) if the cancer was located in the upper part of the rectum [7].

### 2.4. Primary and Secondary Outcomes

The primary endpoint was the peak pressure, which was measured and recorded before operation (T1) and at 30 min after pneumoperitoneum (T2).

The secondary outcomes include plateau pressure, dynamic compliance, arterial blood analysis (e.g., PaCO_2_, pH, oxygen saturation of pulse oximeter (Spo_2_), and the ratio of arterial oxygen partial pressure to fractional inspired oxygen (PaO_2_/FiO_2_, oxygen index)), and hemodynamic measures (e.g., MAP and heart rate (HR)). Plateau pressure and dynamic compliance were measured at T1 and T2. The arterial blood analyses were collected at T1, T2, and 24 h (T3) and 72 h (T4) postoperation. During the surgery, the hemodynamic measures were monitored every 30 min.

The surgery-related adverse events, including pneumothorax, vomiting, delayed recovery, dysphoria, and postoperative cognitive dysfunction (POCD), were monitored and recorded.

### 2.5. Statistical Analysis

Assuming a difference of 7 cmH_2_O and a standard deviation of 4 cmH_2_O [8], 30 patients per group would provide the trial with 90% power at a 5% significance level to show a significant difference in the primary outcome between at least one of hypercapnia groups and control group. Continuous variables with normal distribution are presented as mean ± SD and categorical variables were summarized and expressed as proportions. Baseline characteristics were compared with the use of the chi-square test or Fisher's exact test for categorical variables and by Analysis of Variance (ANOVA) for continuous variables, followed by *post hoc* analysis. A two-way repeated ANOVA and *post hoc* analysis were performed to compare the distribution of primary and secondary outcomes among the three groups.

All statistical analyses were performed with the SPSS statistical software program package (SPSS version 20.0 for Windows, SPSS Inc., Chicago, Illinois, USA). All tests were 2-sided and a *P* value of less than 0.05 was considered significant.

## 3. Results

### 3.1. Baseline and Surgical Characteristics

As shown in Table 1, there is no significant difference in the baseline characteristics (age, gender distribution, BMI, ASA classification, clinical stage, and distance of tumor from anal verge) and surgical features (PaCO_2_, duration of operation, total volume of fluids infused, and blood loss) (all *P* > 0.05).

**Figure 1:**
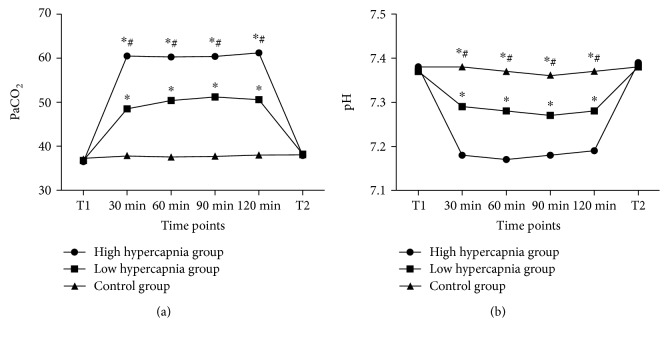
The ventilatory parameters during the pneumoperitoneum. The PaCO_2_ levels (a) were significantly higher in the high hypercapnia group than in the low hypercapnia group and control group, whereas the pH values (b) were significantly lower in the high hypercapnia group. T1: before operation; T2: 30 min after pneumoperitoneum. ^∗^Compared with control group; ^#^compared with low hypercapnia group.

During the pneumoperitoneum, the values of PaCO_2_ were significantly higher in the high hypercapnia group than in the low hypercapnia group and control group (Figure 1; all *P* < 0.01), whereas the pH values were significantly lower in the high hypercapnia group (Figure 1; all *P* < 0.01).

### 3.2. Primary and Secondary Outcomes

Table 2 demonstrates that the differences in the peak pressure, plateau pressure, and dynamic compliance before operation were not statistically significant among the three groups (all *P* > 0.05). At T2, there are significant differences among the three groups in these three ventilator parameters (all *P* < 0.01); *post hoc* analyses showed that both peak pressure and plateau pressure were significantly lower in the high hypercapnia group compared with the low hypercapnia group and control group, while the dynamic compliance was significantly higher in the high hypercapnia group. Meanwhile, the differences between low hypercapnia group and control group were also statistically significant.

**Figure 2:**
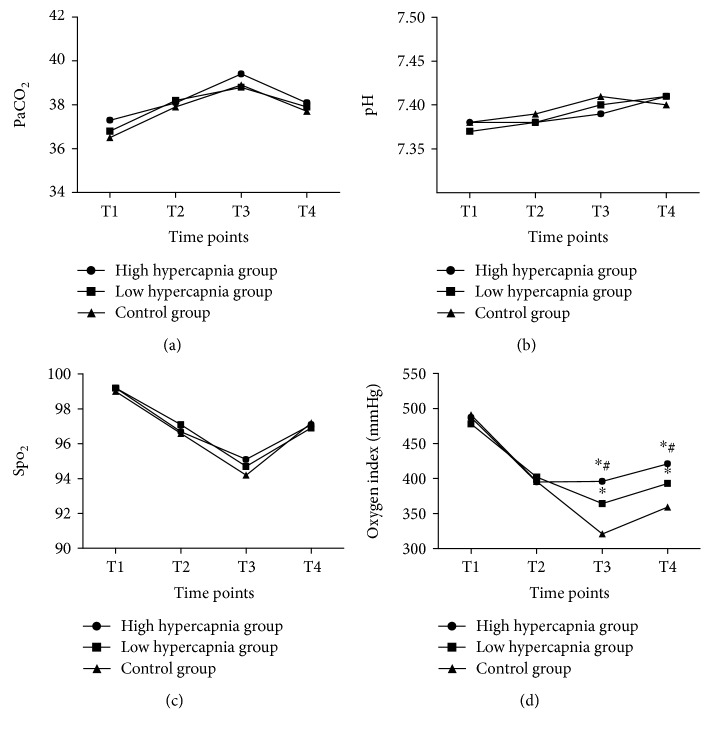
Analysis of arterial blood samples. The difference in the levels of arterial blood PaCO_2_ (a), pH (b), and Spo_2_ (c) was not significant at each time point in the three groups; the ratio of arterial oxygen partial pressure to fractional inspired oxygen (PaO_2_/FiO_2_, oxygen index) in the high hypercapnia group was significantly higher than that in the low hypercapnia group and control at T3 and T4 (d). T1: before operation; T2: 30 min after pneumoperitoneum; T3: 24 h postoperation; T4: 72 h postoperation. ^∗^Compared with control group; ^#^compared with low hypercapnia group.

The oxygen index value (Figure 2(d)) was significantly higher in the high hypercapnia group compared with low hypercapnia group and control group at T3 and T4 (all *P* < 0.01). In terms of PaCO_2_ (Figure 2(a)), Spo_2_ (Figure 2(b)), and pH (Figure 2(c)), there is no significant difference in their values among the three groups from T1 to T4.

**Figure 3:**
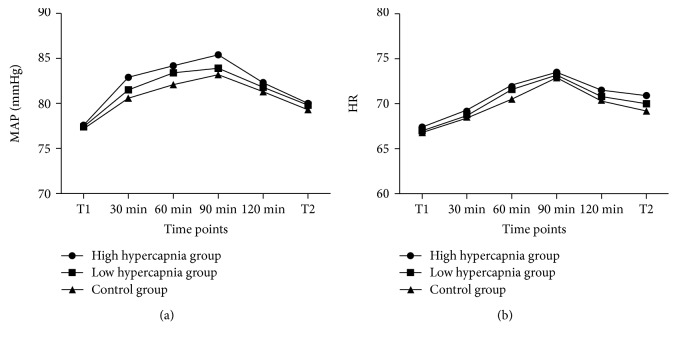
Analysis of hemodynamic measures. The difference in the levels of MAP (a) and HR (b) was not significant at each time point in the three groups. T1: before operation; T2: 30 min after pneumoperitoneum.

The values in the MAP (Figure 3(a)) and HR (Figure 3(b)) in the high hypercapnia group seem to be slightly higher compared with the other groups, but the differences were not significant.

No patients required a blood transfusion or developed adverse surgical events throughout the observation period.

## 4. Discussion

Recent experimental and clinical studies show that permissive hypercapnia is an efficient ventilatory strategy as it can avoid airway hypertension-induced barotraumas and circulatory disorders, while ensuring proper gas exchange to maintain body tolerance [9]. However, the effects of permissive hypercapnia on relatively long-term surgeries are rarely reported. The current study suggests that high PaCO_2_ was associated with a lower airway pressure and a higher dynamic compliance compared to low PaCO_2_ and control group during the laparoscopic surgery. Moreover, oxygen index values were higher in the high hypercapnia group. No significant difference in the pH, Spo_2_, MAP, heart rate, and adverse events was found in the three groups.

CO_2_ pneumoperitoneum increases intra-abdominal pressure, then respiratory mechanics may be altered by decreasing lung volume and increasing airway pressure. Jo et al. showed that during pneumoperitoneum, peak and plateau airway pressures increase by more than 50% and lead to approximately 50% decreases in dynamic and static lung compliances for patients undergoing laparoscopic low anterior resection [10]. In our study, the effects of permissive hypercapnia on peak and plateau airway pressures and dynamic compliance are likely to be caused by the stimulation of sympathetic nerves by carbon dioxide. It is suggested that the stimulation of sympathetic nerves could mediate the relaxation of airway smooth muscles and then expand the airway, thereby decreasing the resistance of ventilation and increasing dynamic compliance [8].

During laparoscopic surgery, atelectasis might bring about abnormal intraoperative gas exchange due to surgery-related inflammation, leading to postoperative lung dysfunction even in patients without preexisting lung injury [11]. A lower oxygen index may be a reflection of a persistent lung dysfunction [12]. In our study, the increased oxygen index in the hypercapnia groups may be caused by the Bohr Effect, in which increases in the carbon dioxide partial pressure of blood resulted in a lower affinity of hemoglobin for oxygen.

There is no observed adverse event, indicating that permissive hypercapnia should be a safe procedure for the management of patients with rectal carcinoma undergoing laparoscopic surgery.

In conclusion, permissive hypercapnia with a higher PaCO_2_ level of 56-65 mmHg was able to improve respiratory function after laparoscopic surgery in rectal cancer patients.

## Figures and Tables

**Table 1:** Patients' baseline and surgical characteristics.

	High hypercapnia group (*n* = 30)	Low hypercapnia group (*n* = 30)	Control group (*n* = 30)	*P* value
Age, years	60.2 ± 6.4	60.7 ± 7.9	61.5 ± 8.2	0.79
Male, *n* (%)	18 (60.0%)	17 (56.7%)	18 (60.0%)	0.96
BMI (kg/m^2^)	24.9 ± 1.7	25.0 ± 1.7	25.4 ± 1.6	0.35
American Society of Anesthesiologists classification, *n* (%)				
II: mild systemic disease	20 (66.7%)	19 (63.3%)	19 (63.3%)	0.95
III: severe systemic disease	10 (33.3%)	11 (36.7%)	11 (36.7%)	
Clinical stage, *n* (%)				
I	8 (26.7%)	7 (23.3%)	8 (26.7%)	0.99
II	12 (40.0%)	13 (43.3%)	13 (43.3%)	
III	10 (33.3%)	10 (33.3%)	9 (30.0%)	
Distance of tumor from anal verge, *n* (%)				
Upper rectum: 10 to 15 cm	9 (30.0%)	10 (33.3%)	9 (30.0%)	0.95
Middle rectum: 5 to <10 cm	11 (36.7%)	12 (40.0%)	9 (30.0%)	
Lower rectum: <5 cm	10 (33.3%)	8 (26.7%)	11 (36.7%)	
PaCO_2_ (mmHg)	37.9 ± 3.8	38.2 ± 3.7	37.6 ± 4.1	0.83
Duration of operation (min)	136.1 ± 19.0	139.0 ± 18.5	140.3 ± 17.7	0.66
Total volume of fluids infused (ml)	1628 ± 158	1674 ± 152	1654 ± 154	0.52
Blood loss (ml)	197.1 ± 45.1	197.2 ± 37.8	188.5 ± 37.8	0.63

**Table 2:** The ventilatory parameters in the three groups at T1 and T2.

	High hypercapnia group (*n* = 30)	Low hypercapnia group (*n* = 30)	Control group (*n* = 30)	*P* value
Peak pressure (cmH_2_O)				
T1	16.7 ± 2.2	17.4 ± 1.7	17.3 ± 2.3	0.36
T2	22.4 ± 3.6^∗^^#^	26.5 ± 3.1^∗^	30.2 ± 3.9	<0.01
Plateau pressure (cmH_2_O)				
T1	15.0 ± 1.8	15.1 ± 2.4	14.8 ± 1.6	0.81
T2	20.1 ± 2.9^∗^^#^	23.4 ± 3.1^∗^	27.0 ± 3.5	<0.01
Dynamic compliance (ml/cmH_2_O)				
T1	55.7 ± 5.9	55.9 ± 5.7	57.1 ± 5.4	0.58
T2	45.6 ± 5.7^∗^^#^	42.3 ± 4.5^∗^	36.6 ± 6.7	<0.01

T1 = before operation; T2 = 30 min after pneumoperitoneum. ^∗^Compared with control group; ^#^compared with low hypercapnia group.

## Data Availability

The data used to support the findings of this study are available from the corresponding author upon request.
